# Novel Cross-Border Approaches to Optimise Identification of Asymptomatic and Artemisinin-Resistant Plasmodium Infection in Mobile Populations Crossing Cambodian Borders

**DOI:** 10.1371/journal.pone.0124300

**Published:** 2015-09-09

**Authors:** Hannah M. Edwards, Sara E. Canavati, Chandary Rang, Po Ly, Siv Sovannaroth, Lydie Canier, Nimol Khim, Didier Menard, Ruth A. Ashton, Sylvia R. Meek, Arantxa Roca-Feltrer

**Affiliations:** 1 Malaria Consortium, Phnom Penh, Cambodia; 2 National Center for Parasitology, Entomology and Malaria Control (CNM), Phnom Penh, Cambodia; 3 Malaria Molecular Epidemiology Unit, Institut Pasteur in Cambodia, Phnom Penh, Cambodia; Ehime University, JAPAN

## Abstract

**Background:**

Human population movement across country borders presents a real challenge for malaria control and elimination efforts in Cambodia and its neighbouring countries. To quantify *Plasmodium* infection among the border-crossing population, including asymptomatic and artemisinin resistant (AR) parasites, three official border crossing points, one from each of Cambodia's borders with Thailand, Laos and Vietnam, were selected for sampling.

**Methods and Findings:**

A total of 3206 participants (of 4110 approached) were recruited as they crossed the border, tested for malaria and interviewed. By real-time polymerase chain reaction (RT-PCR), 5.4% of all screened individuals were found to harbour *Plasmodium* parasites. The proportion was highest at the Laos border (11.5%). Overall there were 97 *P*. *vivax* (55.7%), 55 *P*. *falciparum* (31.6%), two *P*. *malariae* (1.1%) and 20 mixed infections (11.5%). Of identified infections, only 20% were febrile at the time of screening. Of the 24 *P*. *falciparum* samples where a further PCR was possible to assess AR, 15 (62.5%) had mutations in the K13 propeller domain gene, all from participants at the Laos border point. Malaria rapid diagnostic test (RDT) pLDH/HRP-2 identified a positivity rate of 3.2% overall and sensitivity compared to RT-PCR was very low (43.1%). Main individual risk factors for infection included sex, fever, being a forest-goer, poor knowledge of malaria prevention methods and previous malaria infection. Occupation, day of the week and time of crossing (morning vs. afternoon) also appeared to play an important role in predicting positive cases.

**Conclusions:**

This study offers a novel approach to identify asymptomatic infections and monitor AR parasite flow among mobile and migrant populations crossing the borders. Similar screening activities are recommended to identify other hot borders and characterise potential hot spots of AR. Targeted “customised” interventions and surveillance activities should be implemented in these sites to accelerate elimination efforts in the region.

## Introduction

In recent years across the Greater Mekong Subregion, malaria initiatives have increasingly switched from malaria control to a focus on achieving malaria elimination. In Cambodia, the National Centre for Parasitology, Entomology and Malaria Control (CNM) has given itself the goal of achieving complete elimination of malaria by 2025 [1]. However, the region has a high volume of population movement, both within and between countries, causing a great hindrance to achieving this goal because of importation of infection, spread of drug resistance, and challenges in providing malaria services to populations at higher risk of malaria.

When populations move from areas of high to low transmission they hinder control and elimination of malaria by importing infections and facilitating spread of drug resistance [2, 3]. It is the circulation of populations (short-term, cyclical movement) that results in importation of new infections which can then act as a source of local transmission [4, 5]. Cross-border movement of populations has been attributed to maintenance of ‘hot spots’ of high transmission along international borders [6], whilst high volume of movement has been associated with spread of drug resistance, such as that historically seen along the border of Thailand and Cambodia [7]. The movement of populations contributes to both development of drug resistance and the dispersal of drug resistant parasites because mobile populations often experience delays in receiving diagnosis and treatment, have improper health-seeking behaviour or self-medicate, and are subject to lower levels of surveillance [3, 8, 9]. High frequency of cross-border movement is evident between Cambodia and its neighbours: Thailand, Laos and Vietnam [7, 10, 11]; and frequency of border-crossing among Cambodian people has previously been associated with malaria infection [12].

With the upcoming ASEAN Economic Community agreement to be put into effect in 2015, allowing free movement of goods, services and labour between ASEAN countries [13], and the fact that Cambodia's young population [14] will increasingly be entering the work force in coming years, population movement is expected to rise as people will look further afield to bring in an income [7].

Despite this, there have been no substantial efforts to quantify the extent of *Plasmodium* infection within border-crossing populations, and limited activities to try and identify infected persons and target them for treatment. The only ongoing border screening activity taking place in Cambodia thus far has been conducted at Phsar Prom border point on the Cambodia-Thai border, whereby febrile individuals are tested for malaria. However, it is reasonable to suspect that many febrile individuals would not usually make the long, hot journey across the border, resulting in a limited number of symptomatic malaria patients identified. Most importantly, the current protocol is also failing to identify all asymptomatic infections which are likely to significantly contribute to the spread of artemisinin resistant (AR) parasites.

Asymptomatic infections have been recognised as making up a large proportion of infections in both high transmission settings (due to acquisition of immunity) and are increasingly reported in low transmission settings [15], where infections with low and sub-microscopic parasite densities are highly prevalent [16, 17]. Therefore, an even greater hindrance to elimination is created because more infections might not be able to be detected by conventional means, mostly passive surveillance activities [18–20].

Furthermore, although prevalence of malaria evaluated by microscopy in Cambodia has fallen greatly to only 0.9% in the general population according to the Cambodia Malaria Survey 2010 (CMS2010), this is concentrated in certain high risk groups such as mobile populations and forest-goers (1.5% and 2.5% respectively) [21]. Since forested regions are concentrated along borders and much of the cross-border movement is from the migrant worker population, it is reasonable to suspect that malaria prevalence in border-crossers may be as high as that seen in these most high-risk groups. At Phsar Prom, a border crossing point between Cambodia and Thailand, with an average of 1860 crossers per day (personal communication with border officials); the worst-case scenario with a 2.5% prevalence could mean up to 47 border-crossers per day, or 16,973 per year are infected with malaria.

Additionally, surveillance of AR within Cambodia is not currently linked to any cross-border activity. Surveillance of AR in border areas is important because drug resistance has historically developed in border regions, and monitoring its spread is vital for containment and prevention of spread to the African continent, where AR would prove catastrophic [22, 23].

In response to the need to document the spread of malaria across borders, this study aimed to quantify the extent of malaria infection, including asymptomatic infection and AR parasites, in border crossing populations at specific sites on each of the Cambodian borders with Thailand, Laos and Vietnam, and to potentially identify “hot borders” in the country. Investigation of *a priori* risk factors for infection was conducted and hence provided a feasible approach to identify both symptomatic and asymptomatic malaria infection at Cambodian borders.

## Methods

### Ethical approval

In addition to initial verbal consent for participating in the study, individual written consent (documented by signature and/or finger print) was documented in the information sheets and individual questionnaires. Participant tests and interviews were linked according to a unique identification number system. The National Malaria Control Programme agreed to these consent procedures and The Cambodian National Ethics Committee for Health Research approved the overall study protocol after institutional review (approval number 0101-NECHR, 7th June 2013) prior to data collection.

### Study Area

**Fig 1:**
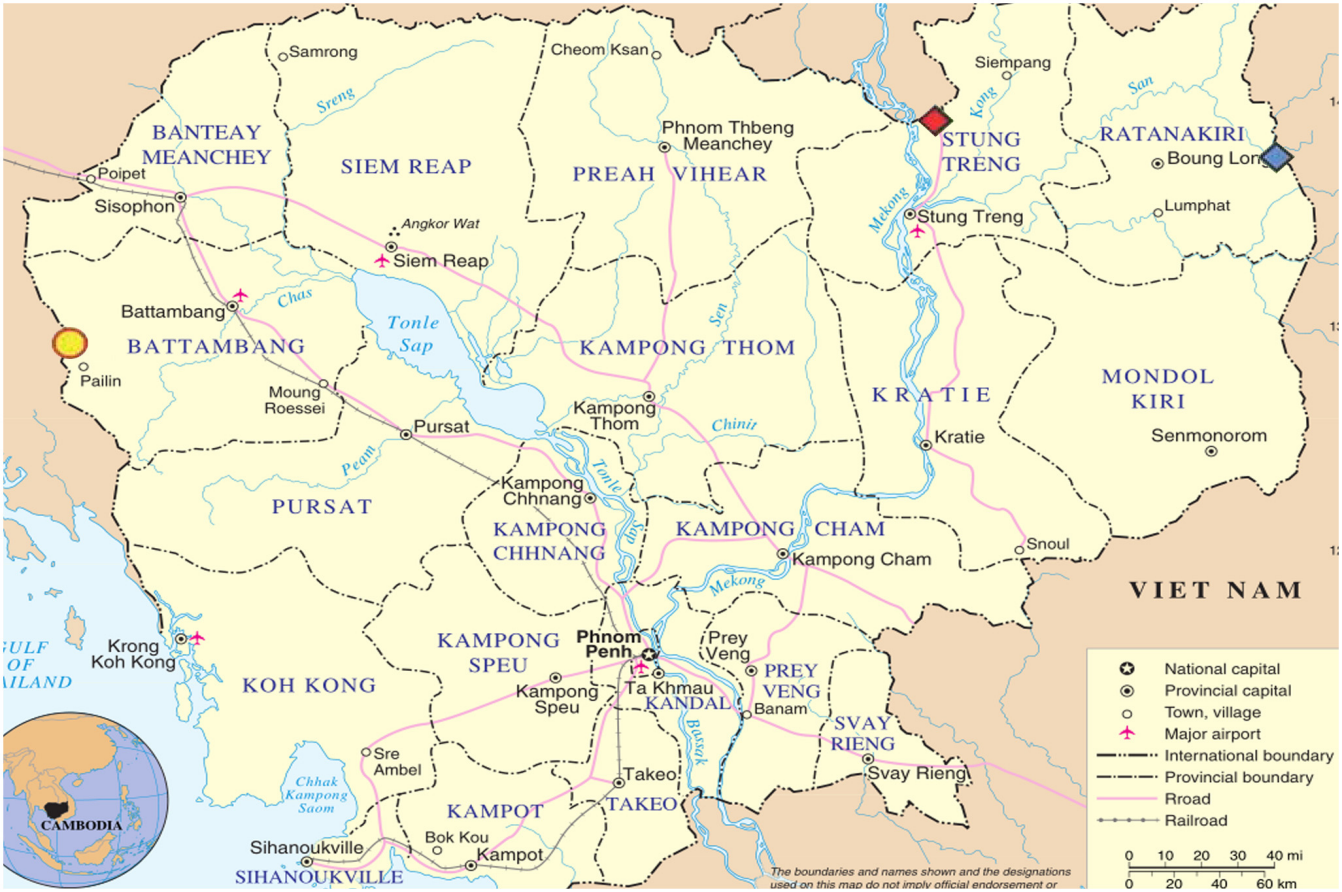
Map of study region and border crossings where cross-border surveillance was implemented. Phsar Prom (Thailand border); Trapaing Kreal (Laos border); O’yadao (Vietnam border).

In order to characterise the types of populations crossing Cambodian borders, quantify the amount of *Plasmodium* infection in these populations and investigate risk factors for infection, three official border crossing points were selected for sampling: Phsar Prom, Pailin province (Cambodia-Thailand border); Trapaing Kreal, Stung Treng province (Cambodia-Laos border); and O’yadao, Rattanakiri province (Cambodia-Vietnam border) (Fig 1). One border crossing point was chosen from each of Cambodia's borders with its different neighbouring countries in order to investigate potential heterogeneity in border-crossing populations and enable identification of hot borders. From each border, the specific site was chosen due to apparent high frequency of use and logistical advantages.

### Sample Size and population

Sample size was calculated at 3000 (e.g. 1000 samples per site), sufficient to provide enough precision (alpha = 0.05, power = 0.9) to capture at least 3% prevalence based on real time polymerase chain reaction (RT-PCR) analysis (the PCR prevalence of parasitaemia found in the Cambodia Malaria Survey 2010; personal communication) and with enough power to explore all secondary objectives and capture seasonal heterogeneity.

Participants were recruited as they crossed the border. Any person crossing the border was eligible for participation and efforts were made to capture those both entering and exiting Cambodia, those on foot and in vehicles, and people of any nationality. All ages were eligible, though for infants and young children, parental assent was taken and parents were interviewed on behalf of the child.

Number of refusals, defined as those that refused to participate after being approached and explained the purpose of the study, were recorded along with reasons for refusal where possible. Toward the end of the study period, it was clear refusals made up a high proportion of those approached and so field teams attempted to record some observatory data on the refusal population including sex, approximate age group and mode of transport. Mode of transport was taken as a proxy for socioeconomic status (SES), with people in cars deemed as high SES and on foot as low SES.

### Recruitment and consent

From mid-August 2013 until mid-February 2014, booths were set up at each of the three selected official border points with malaria health education messages in English, Khmer and the language of the respective bordering country. Booths were set up at the Cambodian side of each border and designed to attract participants and offer shelter, with a table and set of chairs for use while conducting the interviews. Individuals were approached and encouraged to participate as they crossed the border. Participants were invited into the booth, fully informed of the procedure and purpose of the study, and individual consent was gathered.

Sampling at each site was scheduled so as to be able to investigate patterns of infection related to the time of crossing. On the Laos and Vietnam borders, field teams worked five days per week from 7am until 5pm when the border crossing was open, and including where possible four days from Monday-Friday, and either Saturday or Sunday. On the Thai border, due to the high volume of use by border crossers, field teams initially followed the same schedule but then reduced to two days per week, either in the morning or afternoon from one day Monday-Friday and one of either Saturday or Sunday, chosen at random.

### Tests and blood samples

Participant temperature was taken using an infrared thermometer in order to test for presence of fever, defined as a temperature of ≥37.5°C. Malaria diagnosis was determined by rapid diagnostic test malaria antigen pLDH/HRP-2 combo (RDT), brand SD BIOLINE Malaria Ag P.f/P.v, able to distinguish between *P*. *falciparum*, *P*. *vivax* and *P*. *falciparum*/*P*. *vivax* mixed infection. A single finger prick was used to complete the RDT and to produce a dry-blood spot (DBS) filter paper. DBS samples were sent to Institut Pasteur du Cambodge (IPC), Phnom Penh, for RT-PCR analysis. Samples were screened for the presence of *Plasmodium* DNA using a qualitative RT-PCR assay targeting the *Plasmodium cytochrome b* gene, and the positive samples were analysed for *Plasmodium* species using 4 RT-PCR assays specifically amplifying *P*. *falciparum*, *P*. *vivax*, *P*. *ovale and P*. *malariae* [24]. Positive *P*. *falciparum* samples as indicated by RT-PCR were also tested for the presence of mutations in the *K13 propeller domain* gene (*PF3D7_1343700)*, recently associated to AR [25].

### Interviews

While waiting for the RDT result, participants were interviewed according to a questionnaire designed to gather information on *a priori* risk factors for *Plasmodium* infection, including demographic factors such as age, sex and occupation; travel habits such as frequency of border-crossing; and factors relating to knowledge and prevention of malaria. The definition of a ‘forest-goer’ was kept in line with the previous CMS2010 to allow comparison with known risk groups (forest-goer defined as someone who had slept overnight in the forest at least once in the previous 6 months) [21]. Participants were interviewed in a way that was clear to the participant and answers were marked clearly on the questionnaire. Once complete, participants were informed of their RDT result. If positive, they were treated according to Cambodian national treatment guidelines.

### Data analysis

Each questionnaire was double entered into an Epidata 3.1 (Epidata Association, Odense, Denmark) database before being cleaned and analysed in Stata (Version 12, StataCorp, College Station, Texas) [26].

Univariate logistic regression was performed to identify risk factors separately for the outcomes of *Plasmodium falciparum* and *Plasmodium vivax* infection as determined by RT-PCR analysis. All variables with a p-value of less than 0.05 from a likelihood ratio test in univariate analyses were entered into a multivariate logistic regression model and stepwise backwards elimination was used to determine a final list of main risk factors for infection. This process was repeated for symptomatic and asymptomatic cases separately, as well as separately for each border point. Age and sex were to be included in the model whether significant or not.

#### RDT vs. RT-PCR analysis

As a more sensitive diagnostic tool, RT-PCR results only were used as the primary outcome in the multivariate modelling described above. Individual RT-PCR and RDT results were compared in order to determine number of false positives and false negatives that would have resulted from the use of RDT alone, and thus evaluate effectiveness of the testing procedure in the field and potential impact on any subsequent surveillance activity at border points.

## Results

### Study population and refusals

Between August 2013 and March 2014, a total of 3,206 participants were included in the study (1,007 from the Vietnam border; 1,144 from the Laos border, and 1,055 from the Thai border). Of people that were approached (4110) to take part, 904 (22%) refused. Main reasons for refusal (multiple answers possible) included not having enough time (51.6%), not perceiving themselves to be at risk of malaria and thus did not require testing (40.6%), being scared to give blood (34.2%), or having an apparent language or cultural barrier (23.9%).

Observational data on refusals proved difficult to gather and thus is limited to small numbers as shown in Table 1. Although small, there appeared to be an indication of differences between the populations. There appeared to be no difference by sex with the study population, but there was a difference with age (more 15–40 year olds and fewer >40 year olds in the refusal population); nationality (more Thai, Vietnamese and 'Other' nationalities refused, and fewer Cambodian and Laos refused) (Table 1). It was also observed that there was a population of border crossers unable to be approached at all. These were verbally reported by field teams to predominantly be people travelling in cars, trucks and buses, and thus assumedly of a higher SES compared to those crossing on foot.

**Table 1:** Key demographic indicators of the study and refusal populations approached to participate in the study, summarised across all three border points.

		Study population		Refusal population	
		n	% (95% CI)	n	% (95% CI)
**Total population**		3206		904	
**Total interviewed**		3206		162	
**Sex** ^**1**^	**Male**	2171	67.7 (66.1–69.3)	99	63.9 (56.3–71.5)
	**Female**	1034	32.3 (30.7–33.9)	56	36.1 (28.5–43.7)
**Age (years)** ^**2**^	**<15**	219	6.8 (5.9–7.7)	16	10.3 (5.5–15.1)
	**15–40**	2237	69.8 (68.2–71.4)	133	85.8 (80.3–91.3)**
	**>40**	749	23.4 (21.9–24.9)	6	3.9 (0.9–6.9)**
**Nationality** ^**3**^	**Cambodian**	2614	81.6 (80.3–82.9)	71	49.0 (40.9–57.1)**
	**Laos**	356	11.1 (10.0–12.2)	4	2.8 (0.1–5.5)*
	**Thai**	23	0.7 (0.4–1.0)	8	5.5 (1.8–9.2)**
	**Vietnamese**	147	4.6 (3.9–5.3)	42	29.0 (21.6–36.4)**
	**Other**	64	2.0 (1.5–2.5)	20	13.8 (8.2–19.4)**

Difference between study pop^n^ and refusal pop^n^ proportions:

*p<0.05,

**p<0.001

CI = Confidence Interval

^1^Sex missing from 1 individual in the study population and 7 refusals

^2^Age missing from 1 individual in study population and 7 refusals

^3^Nationality missing from 2 individuals in study population and 17 refusals

In terms of the study population (Table 2), there was a significantly higher proportion of males at the Vietnam (76.8%, 95% CI 74.2–79.4) and Laos borders (78.0%, 75.6–80.4) compared to the Thailand border (48.1%, 45.0–51.1). The Laos border had a much higher proportion of fever cases (13.5%, 11.5–15.4) compared to the Vietnam (3.9%, 2.7–5.1) and Thailand borders (1.2%, 0.6–1.9). While the majority of border-crossers were either agricultural workers or other groups believed to be at low risk of malaria (including students, teachers, sales people, etc.), there were more security/armed forces individuals (including security personnel, army officers and police officers) crossing at both Vietnam (7.1%, 5.5–8.7) and Laos (6.7%, 5.2–8.1) borders compared to the Thailand border (0.4%, 0–0.8), and more manual labour workers (including forestry workers, construction workers, etc.) on the Laos border (8.7%, 7.0–10.3) compared to the other two. The Laos border also had greater proportion of: (i) people with a previous episode of malaria, (ii) forest-goers and, (iii) people who knew fewer than two methods of protection against malaria (see Table 2). The majority of sampled individuals at the Thailand border cross more than once per week (67.2%, 64.4–70.0), while at the Laos border majority crossed less than once per week (69.6%, 66.9–72.3).

**Table 2:** Characteristics of the study population by border site, including demographic indicators, frequency of border crossing, history and knowledge of malaria, and key risk behaviours hypothesised to be associated with malaria.

Variable		Thailand		Vietnam		Laos	
		n	% (95% CI)	n	% (95% CI)	n	% (95% CI)
**n**		1055		1007		1144	
**Sex**	**Male**	507	48.1 (45.0–51.1)	773	76.8 (74.2–79.4)	891	78.0 (75.6–80.4)
	**Female**	548	51.9 (48.9–55.0)	234	23.2 (20.6–25.9)	252	22.1 (19.6–24.5)
**Age (years)**	**<15**	72	6.8 (5.3–8.4)	93	9.2 (7.4–11.0)	54	4.7 (3.5–6.0)
	**15–40**	792	75.1 (72.5–77.8)	679	67.4 (64.5–70.3)	766	67.0 (64.2–69.7)
	**>40**	190	18.0 (15.7–20.4)	235	23.3 (20.7–26.0)	324	28.3 (25.7–30.9)
**Current fever (≥37.5°C)**	**No**	1042	98.8 (98.1–99.4)	968	96.1 (94.9–97.3)	990	86.5 (84.6–88.5)
	**Yes**	13	1.2 (0.6–1.9)	39	3.9 (2.7–5.1)	154	13.5 (11.5–15.4)
**Nationality**	**Cambodia**	1014	96.3 (95.2–97.4)	866	86.0 (83.9–88.1)	734	64.2 (61.4–66.9)
	**Vietnam**	3	0.3 (0–0.6)	141	14.0 (11.9–16.2)	3	0.3 (0–0.6)
	**Thailand**	12	1.1 (0.5–1.8)	0		11	1.0 (0.4–1.5)
	**Laos**	0		0		356	31.1 (28.4–33.8)
	**Other**	24	2.3 (1.4–3.2)	0		40	3.5 (2.4–4.6)
**Occupation Group**	**Security/Armed forces**	4	0.4 (0–0.8)	71	7.1 (5.5–8.7)	76	6.7 (5.2–8.1)
	**Manual Labour**	37	3.5 (2.4–4.6)	55	5.5 (4.1–6.9)	99	8.7 (7.0–10.3)
	**Agricultural**	612	58.2 (55.2–61.2)	522	52.0 (48.9–55.1)	532	46.6 (43.7–49.5)
	**Other**	399	37.9 (35.0–40.9)	356	35.5 (32.5–38.4)	435	38.1 (35.3–40.9)
**Previous malaria episode**	**No**	439	43.7 (40.7–46.8)	607	53.3 (50.3–56.2)	251	23.9 (21.3–26.5)
	**Yes**	465	46.3 (43.2–49.4)	515	45.2 (42.3–48.1)	800	76.1 (73.5–78.7)
	**DK**	100	10.0 (8.1–11.8)	18	1.6 (0.9–2.3)	0	
**Frequency of border crossing**	**≥once per week**	709	67.2 (64.4–70.0)	370	36.9 (33.9–39.9)	210	18.7 (16.4–21.0)
	**<once per week**	238	22.6 (20.0–25.1)	607	60.5 (57.5–63.5)	782	69.6 (66.9–72.3)
	**DK**	108	10.2 (8.4–12.1)	26	2.6 (1.6–3.6)	132	11.7 (9.8–13.5)
**Forest-goer**	**No**	989	93.9 (92.5–95.4)	816	81.7 (79.3–84.1)	683	60.1 (57.3–63.0)
	**Yes**	64	6.1 (4.6–7.5)	183	18.3 (15.9–20.7)	453	39.9 (37.0–42.7)
**Knowledge of malaria prevention**	**<2 methods**	671	63.6 (60.7–66.5)	514	51.0 (48.0–54.1)	986	86.2 (84.2–88.2)
	**2+ methods**	384	36.4 (33.5–39.3)	493	49.0 (45.9–52.1)	158	13.8 (11.8–15.8)

Occupation grouped as: 1) Security personnel/Armed forces also includes police; 2) Manual Labour workers include forest workers, construction workers, hydrology etc.; 3) agricultural workers refers to farm workers, fruit pickers etc.; 4) Other refers to low risk occupations such as teaching, students, sales.

Forest-goer refers to someone who slept overnight in the forest at least once in the previous 6 months.

CI = Confidence Interval

DK = Don’t know

### Malaria infection

During the study period, there were 103 malaria positive infections (3.2% positivity rate (95% CI, 2.6–3.8)) detected by RDT, mainly *P*. *falciparum* (65%), followed by *P*. *vivax* (33%), and mixed *P*. *falciparum*/*P*. *vivax* (1.9%). The site-specific positivity rate by RDT was found to be 0.1% (95%CI, 0–1.0), at the Thai border 1.0% (0.4–1.6) at the Vietnam border and 8.0% (6.5–9.6) at the Laos border (Table 3).

**Table 3:** Proportion of sampled individuals found to have *Plasmodium* infection by RDT and RT-PCR at each border site, including speciation.

	Border Site	N tested	# Positive cases	Positivity rate % (95% CI)	# Pf (%, 95% CI)	# Pv (%, 95% CI)	# Pm (%, 95% CI)	# Mixed Pf/Pv (%, 95% CI)	# Mixed Pv/Pm (%, 95% CI)
**RDT**	**Thailand**	1,055	1	0.1 (0–1.0)	1	0		0	
	**Vietnam**	1,007	10	1.0 (0.4–1.6)	4	6	NA	0	NA
	**Laos**	1,144	92	8.0 (6.5–9.6)	62	28		2	
	**Total**	**3206**	**103**	**3.2 (2.6–3.8)**	**67 (65.0, 55.7–74.4)**	**34 (33.0, 23.8–42.2)**		**2 (1.9, 0–4.7)**	
**RT-PCR**	**Thailand**	1055	7	0.7 (0.2–1.2)	2	5	0	0	0
	**Vietnam**	1007	36	3.6 (2.4–4.7)	4	28	2	1	1
	**Laos**	1143	131	11.5 (9.6–13.3)	49	64	0	18	0
	**Total**	**3205**	**174**	**5.4 (4.6–6.2)**	**55 (31.6, 24.6–38.6)**	**97 (55.7, 48.3–63.2)**	**2 (1.1, 0–2.7)**	**19 (10.9, 6.2–15.6)**	**1 (0.6, 0–1.7)**

Pf = *Plasmodium falciparum*, Pv = *Plasmodium vivax*, Pm = *Plasmodium malariae*

RT-PCR analysis was conducted on 3205 participant samples (Table 3). There were 174 positive infections identified, and species differed to that identified by RDT with the majority being *P*. *vivax* infection (55.7%), fewer *P*. *falciparum* (31.6%) mixed *P*. *falciparum/P*. *vivax* (10.9%), *P*. *malariae* (1.1%) and mixed *P*. *vivax/P*. *malariae* (0.6%). Overall positivity rate was 5.4% (95% CI, 4.6–6.2), differing by site: 0.7% (0.2–1.2) at the Thai border, 3.6% (2.4–4.7) at the Vietnam border and 11.5% (9.6–13.3) at the Laos border.

Compared to RT-PCR (gold standard), overall RDT sensitivity was low at only 43.1%, while specificity was high at 99.1%. With *P*. *falciparum* infection only, RDT had a sensitivity of 55.4%. By border point, sensitivity was lowest at the Thailand border where no positive infections by RT-PCR were identified by RDT, though the number of cases was low, and from the Vietnam border point where sensitivity was only 22.2%. Furthermore, the number of *P*. *vivax* and *P*. *falciparum*/*P*. *vivax* mixed infections identified by RDT was much lower than by RT-PCR (Table 3).

Out of all participants, 206 showed signs of fever at the time of testing, with their temperature measured at ≥37.5°C (Table 4). Of these symptomatic participants, 34 tested positive by RDT and 35 by RT-PCR. Of all positive fevers, 28 cases were identified by both tests, giving RDT sensitivity of 80.0% for symptomatic malaria identification. For symptomatic *P*. *falciparum* infection, sensitivity was 82.4%, and RDT identified 20 *P*. *falciparum* infection compared to 17 by PCR.

**Table 4:** Proportion of symptomatic and asymptomatic *Plasmodium* infections identified by RDT and RT-PCR methods for all species of Plasmodium combined and for Pf infection only.

			RDT		PCR	
Border Site	# participants	# Fever (≥37.5°C)	# symptomatic *Plasmodium* infections (Pf infection only)	# asymptomatic *Plasmodium* infections (Pf infection only)	# symptomatic *Plasmodium* infections (Pf infection only)	# asymptomatic *Plasmodium* infections (Pf infection only)
**Thailand**	1,055	13	0 (0)	1 (1)	0 (0)	7 (2)
**Vietnam**	1,007	39	4 (1)	6 (3)	4 (1)	32 (4)
**Laos**	1,144	154	30 (19)	62 (45)	31 (16)	100 (51)
**Total all species (%, 95% CI)**	**3206**	**206**	**34 (33.0, 23.8–42.2)**	**69 (67.0, 57.8–76.2)**	**35 (20.1, 14.1–6.1)**	**139 (79.9, 73.9–85.9)**
***Total Pf (%*, *95% CI)***			***20 (29*.*0*, *18*.*3–39*.*7)***	***49 (71*.*0*, *60*.*3–81*.*7)***	***17 (23*.*0*, *13*.*4–32*.*6)***	***57 (77*.*0*, *67*.*4–86*.*6)***
**Percentage of all species infection detected as Pf infection**			**58.8 (42.3–75.3)**	**71.0 (60.3–81.7)**	**48.6 (32.0–65.2)**	**41.0 (32.8–49.2)**

Pf = *Plasmodium falciparum*, CI = Confidence Interval, RDT = Rapid Diagnostic Test, PCR = Polymerase Chain Reaction

A very high proportion of *Plasmodium* infections were asymptomatic (Table 4). By RDT, 69 (67%) malaria cases were asymptomatic, and by RT-PCR this was even higher at 139 (79.9%). Concordance between RDT and RT-PCR for asymptomatic infection was very low with only 33.8% sensitivity from the RDT, though specificity was high at over 99%. Considering *P*. *falciparum* infection solely, a similarly high proportion of asymptomatic cases were found both by RDT (71.0%) and PCR and (77.0%). However, the proportion of *P*. *falciparum* asymptomatic infections out of all asymptomatic infections varied significantly, mainly due to the higher number of P. vivax cases identified through PCR (see Table 3) compared to RDT. Based on RDT results, 49 asymptomatic *P*. *falciparum* infections were identified, representing 71% of all-species asymptomatic infection. However, by PCR, 57 P. *falciparum* infections were identified, representing only 41% of all-species asymptomatic infection. The major reason for this discrepancy was the high number of false *P*. *falciparum* positives from RDT compared to PCR.

*Plasmodium falciparum* infections found by RT-PCR were analysed for molecular markers of AR (e.g. mutations in K-13 propeller domain gene). Due to extremely low parasitaemias, 50 of the infections could not be successfully PCR amplified. Of the 24 remaining, 15 (62.5%) had mutations in the targeted sequence and thus showed evidence of AR. Within these 15, there were 10 (66.7%) C580Y, four (26.7%) R539T and one (6.7%) mixed R539T/Wild-type mutations. Although screening for K13 was limited by the small number of samples with sufficient parasite DNA for additional PCR analysis, individuals harbouring mutant parasites appeared to have some common characteristics. All were from the Laos border point and 80% were asymptomatic. The majority were male (93.3%), aged 15–40 years (80%) and of Cambodian nationality (80%), with the remainder (20%) of Laos nationality. Most crossed the border on a weekday (93.3%) and in the morning (66.7%). Most had heard of malaria (93.3%) but had low knowledge of prevention methods (86.7% knew <2 methods of prevention); were forest-goers (73.3%) and had a previous episode of malaria (86.7%). Five of the infections were among forestry workers, four among agricultural workers and another four from security/armed forces personnel.

### Risk factors for malaria infection

Considering that risk factors for malaria are likely to differ by species, analyses were conducted for *P*. *falciparum* and *P*. *vivax* separately as determined by RT-PCR. In this paper, results are presented on analysis of *P*. *falciparum* infection here as this represents the first goal in elimination targets for the region.

Risk factors identified from univariate logistic regression on the outcome of *P*. *falciparum* infection (S1 Table) included being male (Odds Ratio (OR) 11.61, p<0.0001)), having fever (OR 4.64, p<0.0001), having a previous malaria episode (OR 5.41, p<0.0001)), being forest-goers (OR 12.88, p<0.0001)), and seeking testing and treatment at public health facilities or 'other' sources aside from village malaria workers (VMWs) or mobile malaria workers (p = 0.004 and, p = 0.005 for testing and treatment location respectively). The latter group of 'other' included a majority of infections from people who visited the private sector, with only one case administering self-treatment or traditional medicine. Knowledge of two or more methods of prevention was found to be protective (OR 0.48, p = 0.004), and certain occupational groups were associated with higher infection rates: security/armed forces (with this group also including police; OR 8.31), manual labour workers (OR 11.99), and agricultural workers (OR 3.23), when compared to low risk occupations (p<0.0001). Importantly, the specific occupational group with highest *P*. *falciparum* infection was forestry workers (grouped under manual labour workers for analysis) which included 15 *P*. *falciparum* infections from 72 individuals, giving a positivity of 20.83%. Positivity rate of all-species *Plasmodium* infection in this group was 23.6%. Interestingly, all of these forestry worker positive cases reported to live in the same two provinces of Cambodia, suggesting a potential “hot spot” requiring further investigation.

*Plasmodium falciparum* infection also appeared to be higher during the months of August to September, and October to November compared to December-February (p = 0.0002). Length of stay before making a return journey was associated with infection, with a stay of over one week associated with higher infection compared to a stay under one week or returning the same day (p<0.0001), and those crossing less frequently then once per week were associated with higher infection than those crossing more regularly (p<0.0001).

Risk factors that remained within the multivariate logistic model are shown in Table 5. Main risk factors were thus being male, working in manual labour jobs (again mainly due to burden of infection in forestry workers), crossing in the months of August and September, crossing less frequently than once per week, and being a forest-goer. Attempts were made to analyse *P*. *falciparum* infection separately for asymptomatic and symptomatic infection but small numbers limited multivariate analysis. Univariate risk factors remained similar for asymptomatic infection compared to Table 5, while univariate risk factors for symptomatic infection only included forest-goers and occupation group (data not shown). Similarly, numbers were too small to develop meaningful multivariate models to describe risk factors for infection separately by border point.

**Table 5:** Risk factors for *Plasmodium falciparum* infection (both symptomatic and asymptomatic) identified by RT-PCR analysis. Variables listed are those which were retained in the final multivariate model, with both the output from univariate (crude OR) and multivariate (adjusted OR) models presented with odds ratios (OR) and 95% confidence intervals (95% CI), as well as likelihood ratio test p values.

Variable		Positivity rate (%)	Crude OR (95% CI)	p-value	Adjusted OR (95% CI)	p-value
**Sex**	**Male**	3.27	11.61 (3.65–36.94)	<0.0001	3.74 (1.1212.47)	0.01
	**Female**	0.29	1		1	
**Age (years)**	**<15**	0.91	1		1	
	**15–40**	2.59	2.89 (0.70–11.91)	0.1	0.85 (0.19–3.87)	0.9
	**>40**	1.87	2.07 (0.47–9.18)		0.74 (0.15–3.58)	
**Occupation**	**Security/armed forces**	5.96	8.31 (3.25–21.28)		2.31 (0.82–6.51)	
	**Manual Labour**	8.38	11.99 (5.22–27.54)	<0.0001	3.71 (1.46–9.41)	0.02
	**Agricultural**	2.40	3.23 (1.56–6.67)		1.54 (0.68–3.49)	
	**Low risk**	0.76	1		1	
**Calendar period**	**Aug-Sept**	3.22	3.75 (1.82–7.74)		2.84 (1.34–6.02)	
	**Oct-Nov**	2.63	3.05 (1.40–6.62)	0.0002	2.16 (0.9–4.82)	0.01
	**Dec-Feb**	0.88	1		1	
**Frequency of border crossing**	**≥once per week**	0.93	1		1	
	**<once per week**	3.50	3.86 (2.06–7.23)	<0.0001	2.28 (1.18–4.40)	0.03
	**DK**	1.89	2.05 (0.71–5.86)		1.44 (0.49–426)	
**Forest-goer**	**No**	0.68	1	<0.0001	1	<0.0001
	**Yes**	8.14	12.88 (7.44–22.29)		6.06 (3.27–11.24)	

DK = Don’t know

OR = Odds Ratio

CI = Confidence Interval

Forest-goer refers to someone who slept overnight in the forest at least once in the previous 6 months.

Main risk factors retained in the multivariate model for *P*. *vivax* infection (S3 Table) were crossing in the afternoon, crossing less frequently than once per week, having fever, having a previous episode of malaria, and being a forest-goer. Other interesting risk factors found in univariate analyses (S2 Table) included occupational group, with security and armed forces appearing to have highest infection rate. The majority of *P*. *vivax* infections were found in agricultural workers (72 infections) however, being a large group, positivity of *P*. *vivax* infection positivity among agricultural workers was 4.32%. Security and armed forces personnel had the highest percentage of *P*. *vivax* infection with 15 cases and 9.93% *P*. *vivax* positivity. It also appeared as though people crossing on a weekday as opposed to weekend may have higher infection risk, though there was no association with calendar period. Furthermore, those exiting Cambodia had higher odds of *P*. *vivax* infection than those entering.

## Discussion

The study described here showed a feasible approach to optimise screening activities for monitoring AR parasite flow among mobile and migrant populations crossing the borders, identifying hot border crossing points and discriminating them from border points representing less of a threat. The result is a promising method to rapidly discriminate between “hot” *vs* “non-hot” borders and therein enable better targeting of surveillance and intervention efforts by optimising use of resources and the identification of *Plasmodium* infection and AR parasites. To our knowledge, this is a novel approach for the Greater Mekong Subregion, improving access and targeting of the otherwise hard-to-reach mobile and migrant populations as they cross international borders.

The hot border identified in this region was Cambodia's border crossing with Laos in Stung Treng province. This border point had an alarmingly high malaria positivity rate, much higher than that previously found in even the most high-risk groups in Cambodia [21]. The high positivity rate is likely a product of the profile of the border-crossing population frequenting this border point. The population in this border area had higher proportion of people with the main risk factors identified for *P*. *falciparum* infection—high proportion of males, forestry workers, people crossing with a frequency of less than once a week and of forest-goers. In terms of main risk factors for *P*. *vivax* infection, there were also more people with documented fever and a previous episode of malaria. Border crossers at the Laos border also had a poorer knowledge of malaria prevention methods.

The high positivity rate of malaria is of concern here because it will create a hindrance to malaria elimination efforts in Cambodia. Our finding that *Plasmodium* infection in this border-crossing population is substantially higher than other mobile and migrant groups previously surveyed in the national Cambodia Malaria Survey demonstrates that border surveillance may target populations not adequately captured in standard household surveys. In addition, our finding that place of destination and origin of border crossers might represent potential hot spots, promotes the potential future utility of these border surveillance data for case investigations and rapid response systems. Furthermore, the majority of *Plasmodium* infections identified among the border-crossing population are asymptomatic and were not identified by RDT, presenting challenges for future border surveillance activities, but potentially representing an important malaria reservoir and therefore contributing to transmission [27]. The high proportion of AR *P*. *falciparum* infections is particularly worrisome, as Laos has not been formally acknowledged as to be affected by confirmed AR at the time of the study. It would be difficult to determine where these people became infected as seven individuals were travelling from Cambodia and eight were travelling from Laos, yet most (80%) crossed at least monthly. Despite the small number of AR parasites identified, these data provides evidence of the frequent flow of border crossers carrying AR parasites between neighbouring countries, and is the first study to document the flow of AR parasites in the region as well as to offer a feasible strategy to identify potential hot spots of resistance.

It is important to be able to identify and treat these infections. Utilising the risk factors identified here as an identification algorithm at border crossing points offers a potentially efficient method. We have identified that border crossing points can be utilised as an effective point of access to very high-risk populations normally missed by classic surveillance methods. Importantly, the risk factors identified here can be specifically applied to identify either *P*. *falciparum* or *P*. *vivax* infection and there were interesting differences between the risk factors identified for each. For *P*. *falciparum*, infection was more common during the months of August and September as opposed to later in the study, while the same was not seen for *P*. *vivax*. This is likely a result of the seasonality of *P*. *falciparum* infection, while *P*. *vivax* infections identified may be chronic or latent infection rather than newly acquitted. *Plasmodium vivax* infection was higher in people crossing during the afternoon and during the weekday. It is not clear the reason for this, but may offer a potential period for scale-up of any activity, optimising identification of infection with available resources. Also of interest is the lower *P*. *falciparum* infection rates seen in people who had sought treatment or testing for a previous malaria episode with either a VMW/MMW or at a public health facility as opposed to a private health facility. This may highlight the importance of point of access care and dissemination of information, or perhaps a miss-regulation of treatment at private facilities. Further work is needed to investigate the combination of risk factors yielding most *Plasmodium* infections, and assess whether these are significantly different to pick up symptomatic, asymptomatic or AR infections.

Of interesting note for targeting of interventions both at the border and potentially in local communities, is the distribution of infection with certain occupational groups. Security personnel, police and armed forces had high infection risk of *P*. *vivax* and *P*. *falciparum*. Many police and army stations/barracks are located along the border areas and many individuals in these occupational groups reported sleeping overnight in the forest. Manual labour workers had high risk of *P*. *falciparum*, mainly attributable to forestry workers, who were included in the manual labour group and comprised the biggest single occupation group at risk. Forestry workers, security workers, armed forces and police are groups that can potentially be targeted for specific interventions such as insecticide-treated uniforms or hammock nets. Additionally, the forestry workers identified in this study appeared to come from a similar area within Cambodia, highlighting a possible focal point of malaria risk that can be targeted with interventions.

There was very low concordance between RDT and RT-PCR results. Sensitivity of RDT was particularly poor regarding identification of asymptomatic cases and *P*. *vivax* or *P*. *falciparum*/*P*. *vivax* mixed infection. This is in agreement with previous studies that have found poor performance of RDT with *P*. *vivax* identification and identification of low parasitaemia [16, 28, 29]. It is worth noting, however, that RDTs (when combined with fever) were relatively effective in identifying symptomatic infections. However, low parasitaemia is often associated with asymptomatic carriage and with *P*. *vivax* carriage [16, 30] and contributes to malaria transmission [27]. This reflects a problem for future surveillance initiatives as Cambodia approaches elimination, since the proportion of asymptomatic infection and *P*. *vivax* infection will increase, as seen in other low transmission settings [30]. In addition, although numbers of K-13 mutant parasites were low, the majority of AR carriers were found to be asymptomatic emphasising the real need for a more sensitive diagnostic test that can be used at the point of access to rapidly treat and follow up asymptomatic (and potentially AR) infections. Interestingly, RDT detected a greater number of symptomatic *P*.*falciparum* infection compared to PCR (20 vs. 17). This is more likely to have been due to false positive results by RDT rather than false negative PCR, a problem of RDTs that would result in misdiagnosis and use of ACTs at the point-of care[31–34].

The study suffered from some limitations that hampered our ability to assess in detail some risk factors that were significantly associated with *Plasmodium* infections. For example, border points were not selected at random, they were chosen in order to be viable for logistic and operational reasons. Road networks are often of poor quality in border regions of Cambodia and border points are generally situated far from the nearest towns. However, we can assume that the more remote border points will also have fewer people using them and the border crossing population will predominantly be residents close to the border point. Thus, targeting these people may perhaps require a different approach, such as surveillance at the community level as opposed to the border crossing point.

There was also a selection bias in the study population (where participants were predominantly Cambodians) and was likely observed due to the fact of having the screening booths on the Cambodian side of the border. This aspect limited our ability to fully determine effect of nationality, ethnic group, permanent residence and even direction of travel on infection. Foreign nationalities often refused to take part due to language or cultural differences and differences in perception of risk of malaria. Cross-border collaboration and establishment of screening booths on either side of the border points may begin to overcome this cultural difference, allowing booths to be stationed by local field teams and thus be more identifiable and acceptable to respective populations.

In addition, considering that our convenience sample may have missed border crossers with higher socio-economic status, this may have resulted in an overestimation of the true positivity rates of the entire cross-border population, particularly if malaria risk is association with socio-economic factors. We may also have seen a slight overestimation because we failed to identify which individuals had taken anti-malarials within the previous month, potentially resulting in false positives with both RDT and PCR analysis. However, as the main objective of these cross border screening methods is to prioritise high-risk groups, the proposed surveillance approach would be able to identify people most at risk of harbouring malaria parasites, including those carrying asymptomatic infections. Finally, considering that population characteristics differed by border sites it is possible that different risk factors might be identified as predictors of malaria infection. However, low case numbers limited sub-analyses for symptomatic and AR malaria, as well as for study of risk factors by each specific border point. As cross-border surveillance activities continue in the Cambodian-Laos border, subsequent analyses with higher sample sizes would be able to provide further evidence on the predictive factors in these important sub-populations.

Despite these biases, we believe this study has enabled identification of the main risk factors predicting border crossers carrying *Plasmodium* parasites, particularly those with asymptomatic infection. According to our regression models there was a small subset of main risk factors able to identify those at risk of harbouring parasites. These risk factors offer a potential way to target cross-border surveillance for mobile populations, a notoriously hard demographic group to reach. This surveillance can also be linked to programmatic interventions, such as bed net or hammock net distribution, and behaviour change communication to improve knowledge of prevention methods.

Human population movement is a common challenge in malaria control to the South-East Asia region so this method and findings may be generalisable to the rest of the region. Moving forward from this there is a need to implement and test a surveillance algorithm at the Laos hot border. Identified cases need to be followed up, investigated and linked up with a rapid reporting mechanism to national level systems. With the current method, it is impossible to deduce conclusions about source of infection. Now that we have highlighted the usefulness of border screening to target high risk populations, it will be an important next step to conduct case investigation into positive cases to identify possible transmission foci and target responses to the identified hotspots. Furthermore, due to the high amount of population movement, a regional reporting approach could be considered to harmonise case investigations and responses on both sides of the border. Follow-up could also be used to target reactive case detection to identify and test household and community contacts and prevent onward transmission.

Finally, borders are extremely porous in this region and many people are thought to cross via unofficial roads and footpaths into neighbouring countries. We attempted to sample from some of these unofficial border points but localised flooding, security and low frequency of use in our chosen locations limited this. Use of unofficial border points is certainly an area that needs more investigation as it can be assumed that they will be frequented by populations highly vulnerable to infection.

In conclusion, we believe that this approach could be easily implemented to identify additional hot borders in the region. Cross-border collaboration would optimise identification of these and any subsequent interventions to be put in place at appropriate hot spots. Furthermore, evaluation of new point of access diagnostic tests suitable to identify asymptomatic infections is required.

## Supporting Information

S1 Table Univariate risk factors for *Plasmodium falciparum* infection (both symptomatic and asymptomatic) identified by RT-PCR analysis. Variables listed are presented with odds ratios (OR) and 95% confidence intervals (95% CI), as well as likelihood ratio test p values. OR = Odds ratio
CI = Confidence interval
DK = Don't know
VMW/MMW = Village Malaria Worker/Mobile Malaria Worker
HF = Health Facility
Forest-goer refers to someone whom slept overnight in the forest at least once in the previous 6 months.
* For those that had stayed overnight at the journey start, or were planning to stay overnight at their destination
(DOCX)

S2 Table Univariate risk factors for *Plasmodium vivax* infection (both symptomatic and asymptomatic) identified by RT-PCR analysis. Variables listed presented with odds ratios (OR) and 95% confidence intervals (95% CI), as well as likelihood ratio test p values.
OR = Odds ratio
CI = Confidence interval
DK = Don't know
VMW/MMW = Village Malaria Worker/Mobile Malaria Worker
HF = Health Facility
Forest-goer refers to someone whom slept overnight in the forest at least once in the previous 6 months.
* For those that had stayed overnight at the journey start, or were planning to stay overnight at their destination
(DOCX)

S3 Table Main risk factors for *Plasmodium vivax* infection (both symptomatic and asymptomatic) identified by RT-PCR analysis. Variables listed are those which were retained in the final multivariate model, with both the output from univariate (crude OR) and multivariate (adjusted OR) models presented with odds ratios (OR) and 95% confidence intervals (95% CI), as well as likelihood ratio test p values.
OR = Odds ratio
CI = Confidence interval
DK = Don't know
Forest-goer refers to someone who slept overnight in the forest at least once in the previous 6 months.
(DOCX)
