# Supplementary material for: Novel Cross-Border Approaches to Optimise Identification of Asymptomatic and Artemisinin-Resistant Plasmodium Infection in Mobile Populations Crossing Cambodian Borders
Source: PLoS One. 2015 Sep 9;10(9):e0124300. doi: 10.1371/journal.pone.0124300 (PMC4564195; doi:10.1371/journal.pone.0124300)
Supplement: S1 Table — Variables listed are presented with odds ratios (OR) and 95% confidence intervals (95% CI), as well as likelihood ratio test p values. OR = Odds ratio CI = Confidence interval DK = Don't know VMW/MMW = Village Malaria Worker/Mobile Malaria Worker HF = Health Facility Forest-goer refers to someone whom slept overnight in the forest at least once in the previous 6 months. * For those that had stayed overnight at the journey start, or were planning to stay overnight at their destination (DOCX) [file pone.0124300.s001.docx]

**Table S1. Univariate risk factors for *Plasmodium falciparum* infection (both symptomatic and asymptomatic) identified by RT-PCR analysis. Variables listed are presented with odds ratios (OR) and 95% confidence intervals (95% CI), as well as likelihood ratio test p values.**

|  | **Variable** | | **Positivity rate (%)** | **Crude OR**  **(95% CI)** | **p-value** |
| --- | --- | --- | --- | --- | --- |
|  |  |  |  |  |  |
| **Personal/Background** | |  |  |  |  |
|  | **Sex** | **Male** | 3.27 | 11.61 (3.65-36.94) | <0.0001 |
|  |  | **Female** | 0.29 | 1 |  |
|  |  |  |  |  |  |
|  | **Age (years)** | **<15** | 0.91 | 1 | 0.1 |
|  |  | **15-40** | 2.59 | 2.89 (0.70-11.91) |  |
|  |  | **>40** | 1.87 | 2.07 (0.47-9.18) |  |
|  |  |  |  |  |  |
|  | **Nationality** | **Cambodian** | 2.33 | 1 | 0.1 |
|  |  | **Vietnamese** | 0.68 | 0.29 (0.04-2.08) |  |
|  |  | **Thai** | 0 | - |  |
|  |  | **Laos** | 3.37 | 1.46 (0.78-2.74) |  |
|  |  | **Other** | 0 | - |  |
|  |  |  |  |  |  |
|  | **Country of permanent residence** | **Cambodia** | 2.31 | 1 | 0.5 |
|  |  | **Vietnam** | 0.88 | 0.38 (0.05-2.75) |  |
|  |  | **Thailand** | 2.27 | 0.98 (0.13-7.26) |  |
|  |  | **Laos** | 3.25 | 1.42 (0.76-2.67) |  |
|  |  | **Other** | 0 | 1 |  |
|  |  |  |  |  |  |
|  | **Occupation** | **Security/Armed forces** | 5.96 | 8.31 (3.25-21.28) | <0.0001 |
|  |  | **Manual Labour** | 8.38 | 11.99 (5.22-27.54) |  |
|  |  | **Agricultural** | 2.40 | 3.23 (1.56-6.67) |  |
|  |  | **Low-risk** | 0.76 | 1 |  |
|  |  |  |  |  |  |
|  | **Read and write** | **Yes** | 2.14 | 1 | 0.4 |
|  |  | **No** | 2.63 | 1.24 (0.75-2.03) |  |
|  |  |  |  |  |  |
|  | **Fever (≥37.5^o^C)** | **No** | 1.90 | 1 | <0.0001 |
|  |  | **Yes** | 8.25 | 4.64 (2.65-8.14) |  |
|  |  |  |  |  |  |
|  | **Previous malaria episode** | **Yes** | 4.39 | 5.41 (3.05-9.59) | <0.0001 |
|  |  | **No** | 0.84 | 1 |  |
|  |  | **DK** | 1.69 | 2.03 (0.46-8.97) |  |
|  |  |  |  |  |  |
| **Journey information** | |  |  |  |  |
|  | **Time of crossing** | **am** | 1.98 | 1 | 0.02 |
|  |  | **pm** | 3.67 | 1.89 (1.15-3.12) |  |
|  |  |  |  |  |  |
|  | **Day of crossing** | **Mon/Fri** | 2.50 | 2.53 (0.92-6.97) | 0.04 |
|  |  | **Sat/Sun** | 1.00 | 1 |  |
|  |  |  |  |  |  |
|  | **Calendar Period** | **Aug-Sept** | 3.22 | 3.75 (1.82-7.74) | 0.0002 |
|  |  | **Oct-Nov** | 2.63 | 3.05 (1.40-6.62) |  |
|  |  | **Dec-Feb** | 0.88 | 1 |  |
|  |  |  |  |  |  |
|  | **Direction of travel** | **Entering Cambodia** | 2.18 | 1 | 0.6 |
|  |  | **Exiting Cambodia** | 2.44 | 1.12 (0.71-1.78) |  |
|  |  |  |  |  |  |
|  | **Travelling from** | **Cambodia** | 2.50 | 1 | <0.0001 |
|  |  | **Vietnam** | 0.84 | 0.33 (0.08-1.38) |  |
|  |  | **Thailand** | 0.13 | 0.05 (0.01-0.36) |  |
|  |  | **Laos** | 5.49 | 2.27 (1.40-3.66) |  |
|  |  |  |  |  |  |
|  | **Travelling to** | **Cambodia** | 2.14 | 1 | <0.0001 |
|  |  | **Vietnam** | 0.38 | 0.18 (0.05-0.58) |  |
|  |  | **Thailand** | 0.40 | 0.18 (0.02-1.34) |  |
|  |  | **Laos** | 6.09 | 2.97 (1.83-4.80) |  |
|  |  |  |  |  |  |
|  | **Length before return across the border** | **Same day** | 1.29 | 1 | <0.0001 |
|  |  | **≤1 week** | 2.15 | 1.68 (0.84-3.35) |  |
|  |  | **>1 week** | 5.83 | 4.73(2.69-8.32) |  |
|  |  | **DK** | 0.69 | 0.53 (0.07-3.98) |  |
|  |  |  |  |  |  |
|  | **Frequency of border crossing** | **≥once per week** | 0.93 | 1 | <0.0001 |
|  |  | **<once per week** | 3.50 | 3.86 (2.06-7.23) |  |
|  |  | **DK** | 1.89 | 2.05 (0.71-5.86) |  |
|  |  |  |  |  |  |
|  |  |  |  |  |  |
| **Behaviour and malaria knowledge** | | |  |  |  |
|  | **Forest-goer** | **No** | 0.68 | 1 | <0.0001 |
|  |  | **Yes** | 8.14 | 12.88 (7.44-22.29) |  |
|  |  |  |  |  |  |
|  | **Slept under net at journey start*** | **Yes** | 2.92 | 1 |  |
|  |  | **No** | 0 | - |  |
|  |  |  |  |  |  |
|  | **Type of net*** | **Treated** | 4.07 | 1 | 0.001 |
|  |  | **Conventional** | 1.80 | 0.43 (0.25-0.73) |  |
|  |  | **DK** | 0 | - |  |
|  |  |  |  |  |  |
|  | **Plan to sleep under net at destination*** | **Yes** | 3.80 | 1 | 0.02 |
|  |  | **No** | 0.85 | 0.22 (0.05-0.89) |  |
|  |  | **DK** | 6.90 | 1.87 (0.43-8.08) |  |
|  |  |  |  |  |  |
|  | **Have heard about malaria** | **Yes** | 2.56 | 1 | 0.03 |
|  |  | **No** | 0.70 | 0.27 (0.07-1.10) |  |
|  |  | **DK** | 0.74 | 0.28 (0.04-2.05) |  |
|  |  |  |  |  |  |
|  | **Knowledge of malaria prevention** | **<2 methods** | 2.76 | 1 | 0.009 |
|  |  | **2+ methods** | 1.35 | 0.48 (0.27-0.87) |  |
|  |  |  |  |  |  |
|  | **Location of malaria test (for previous episode)** | **VMW/MMW** | 0.56 | 1 | 0.004 |
|  |  | **Public HF** | 4.65 | 8.58 (1.15-63.76) |  |
|  |  | **Other** | 5.44 | 10.12 (1.37-74.69) |  |
|  |  |  |  |  |  |
|  | **Location of malaria treatment (for previous episode)** | **VMW/MMW** | 0.57 | 1 | 0.005 |
|  |  | **Public HF** | 4.82 | 8.77 (1.18-65.09) |  |
|  |  | **Other** | 5.24 | 9.56 (1.29-70.60) |  |
| OR = Odds ratio  CI = Confidence interval  DK = Don't know  VMW/MMW = Village Malaria Worker/Mobile Malaria Worker  HF = Health Facility  Forest-goer refers to someone whom slept overnight in the forest at least once in the previous 6 months.  * For those that had stayed overnight at the journey start, or were planning to stay overnight at theirr destination | | | | | |
