# Supplementary material for: Novel Cross-Border Approaches to Optimise Identification of Asymptomatic and Artemisinin-Resistant Plasmodium Infection in Mobile Populations Crossing Cambodian Borders
Source: PLoS One. 2015 Sep 9;10(9):e0124300. doi: 10.1371/journal.pone.0124300 (PMC4564195; doi:10.1371/journal.pone.0124300)
Supplement: S2 Table — Variables listed presented with odds ratios (OR) and 95% confidence intervals (95% CI), as well as likelihood ratio test p values. OR = Odds ratio CI = Confidence interval DK = Don't know VMW/MMW = Village Malaria Worker/Mobile Malaria Worker HF = Health Facility Forest-goer refers to someone whom slept overnight in the forest at least once in the previous 6 months. * For those that had stayed overnight at the journey start, or were planning to stay overnight at their destination (DOCX) [file pone.0124300.s002.docx]

**Table S2. Univariate risk factors for *Plasmodium vivax* infection (both symptomatic and asymptomatic) identified by RT-PCR analysis. Variables listed presented with odds ratios (OR) and 95% confidence intervals (95% CI), as well as likelihood ratio test p values.**

|  | **Variable** | | **Positivity rate (%)** | **Crude OR**  **(95% CI)** | **p-value** |
| --- | --- | --- | --- | --- | --- |
|  |  |  |  |  |  |
| **Personal/Background** | |  |  |  |  |
|  | **Sex** | **Male** | 4.74 | 3.63 (2.06-6.37) | <0.0001 |
|  |  | **Female** | 1.36 | 1 |  |
|  |  |  |  |  |  |
|  | **Age (years)** | **<15** | 2.28 | 1 | 0.3 |
|  |  | **15-40** | 3.93 | 1.75 (0.70-4.36) |  |
|  |  | **>40** | 3.21 | 1.42 (0.53-3.76) |  |
|  |  |  |  |  |  |
|  | **Nationality** | **Cambodian** | 3.86 | 1 | 0.05 |
|  |  | **Vietnamese** | 0.68 | 0.17 (0.02-1.23) |  |
|  |  | **Thai** | 0 | - |  |
|  |  | **Laos** | 4.21 | 1.09 (0.63-1.90) |  |
|  |  | **Other** | 0 | - |  |
|  |  |  |  |  |  |
|  | **Country of permanent residence** | **Cambodia** | 3.93 | 1 | 0.9 |
|  |  | **Vietnam** | 0 | - |  |
|  |  | **Thailand** | 0 | - |  |
|  |  | **Laos** | 4.07 | 1.04 (0.60-1.80) |  |
|  |  | **Other** | 0 | - |  |
|  |  |  |  |  |  |
|  | **Occupation** | **Security/Armed forces** | 9.93 | 5.85 (2.93-11.55) | <0.0001 |
|  |  | **Manual Labour** | 4.19 | 2.32 (1.02-5.29) |  |
|  |  | **Agricultural** | 4.32 | 2.40 (1.48-3.89) |  |
|  |  | **Low-risk** | 1.85 | 1 |  |
|  |  |  |  |  |  |
|  | **Read and write** | **Yes** | 3.47 | 1 | 0.4 |
|  |  | **No** | 4.17 | 1.21 (0.81-1.80) |  |
|  |  |  |  |  |  |
|  | **Fever (≥37.5^o^C)** | **No** | 3.13 | 1 | <0.0001 |
|  |  | **Yes** | 11.17 | 3.88 (2.40-6.28) |  |
|  |  |  |  |  |  |
|  | **Previous malaria episode** | **Yes** | 7.02 | 5.29 (3.38-8.29) | <0.0001 |
|  |  | **No** | 1.41 | 1 |  |
|  |  | **DK** | 0.85 | 0.60 (0.08-4.46) |  |
|  |  |  |  |  |  |
| **Journey information** | |  |  |  |  |
|  | **Time of crossing** | **am** | 2.87 | 1 | <0.0001 |
|  |  | **pm** | 6.87 | 2.50 (1.70-3.67) |  |
|  |  |  |  |  |  |
|  | **Day of crossing** | **Mon/Fri** | 3.96 | 3.25 (1.32-8.02) | 0.002 |
|  |  | **Sat/Sun** | 1.25 | 1 |  |
|  |  |  |  |  |  |
|  | **Calendar Period** | **Aug-Sept** | 3.68 | 0.99 (0.64-1.53) | 0.9 |
|  |  | **Oct-Nov** | 3.44 | 0.92 (0.57-1.50) |  |
|  |  | **Dec-Feb** | 3.71 | 1 |  |
|  |  |  |  |  |  |
|  | **Direction of travel** | **Entering Cambodia** | 2.68 | 1 | 0.003 |
|  |  | **Exiting Cambodia** | 4.63 | 1.76 (1.20-2.58) |  |
|  |  |  |  |  |  |
|  | **Travelling from** | **Cambodia** | 4.56 | 1 | <0.0001 |
|  |  | **Vietnam** | 2.11 | 0.45 (0.18-1.13) |  |
|  |  | **Thailand** | 0.63 | 0.13 (0.05-0.33) |  |
|  |  | **Laos** | 6.02 | 1.34 (0.88-2.04) |  |
|  |  |  |  |  |  |
|  | **Travelling to** | **Cambodia** | 2.64 | 1 | <0.0001 |
|  |  | **Vietnam** | 3.20 | 1.22 (0.74-2.01) |  |
|  |  | **Thailand** | 0 | - |  |
|  |  | **Laos** | 8.70 | 3.51 (2.30-5.35) |  |
|  |  |  |  |  |  |
|  | **Length** | **Same day** | 3.03 | 1 |  |
|  |  | **<=1 week** | 2.92 | 0.96 (0.56-1.65) |  |
|  |  | **>1 week** | 6.71 | 2.30 (1.48-3.57) | 0.002 |
|  |  | **DK** | 3.45 | 1.14 (0.45-2.92) |  |
|  |  |  |  |  |  |
|  | **Frequency of crossing** | **≥once per week** | 2.25 | 1 |  |
|  |  | **<once per week** | 5.16 | 2.37 (1.54-3.63) | <0.0001 |
|  |  | **DK** | 1.13 | 0.50 (0.15-1.65) |  |
|  |  |  |  |  |  |
|  |  |  |  |  |  |
| **Behaviour and malaria knowledge** | | |  |  |  |
|  | **Forest-goer** | **No** | 1.91 | 1 | <0.0001 |
|  |  | **Yes** | 10.14 | 6.13 (4.17-8.99) |  |
|  |  |  |  |  |  |
|  | **Slept under net at journey start** | **Yes** | 4.51 | 1 | 0.02 |
|  |  | **No** | 1.68 | 0.36 (0.13-0.99) |  |
|  |  |  |  |  |  |
|  | **Type of net** | **Treated** | 6.31 | 1 |  |
|  |  | **Conventional** | 2.61 | 0.40 (0.26-0.61) | <0.0001 |
|  |  | **DK** | 0 | - |  |
|  |  |  |  |  |  |
|  | **Plan to sleep under net at destination** | **Yes** | 4.67 | 1 | 0.2 |
|  |  | **No** | 2.54 | 0.53 (0.23-1.24) |  |
|  |  | **DK** | 6.90 | 1.51 (0.35-6.50) |  |
|  |  |  |  |  |  |
|  | **Have heard about malaria** | **Yes** | 4.03 | 1 | 0.03 |
|  |  | **No** | 1.75 | 0.42 (0.17-1.05) |  |
|  |  | **DK** | 0 | - |  |
|  |  |  |  |  |  |
|  | **Knowledge of malaria prevention** | **<2 methods** | 4.19 | 1 | 0.01 |
|  |  | **2+ methods** | 2.51 | 0.59 (0.38-0.92) |  |
|  |  |  |  |  |  |
|  | **Location of malaria test (for previous episode)** | **VMW/MMW** | 4.52 | 1 | 0.2 |
|  |  | **Public HF** | 6.51 | 1.47 (0.67-3.23) |  |
|  |  | **Other** | 8.42 | 1.94 (0.90-4.19) |  |
|  |  |  |  |  |  |
|  | **Location of malaria treatment (for previous episode)** | **VMW/MMW** | 4.60 | 1 | 0.2 |
|  |  | **Public HF** | 6.68 | 1.49 (0.68-3.26) |  |
|  |  | **Other** | 8.20 | 1.85 (0.86-4.00) |  |
| OR = Odds ratio  CI = Confidence interval  DK = Don't know  VMW/MMW = Village Malaria Worker/Mobile Malaria Worker  HF = Health Facility  Forest-goer refers to someone whom slept overnight in the forest at least once in the previous 6 months.  * For those that had stayed overnight at the journey start, or were planning to stay overnight at their destination | | | | | |
