# Supplementary material for: Novel Cross-Border Approaches to Optimise Identification of Asymptomatic and Artemisinin-Resistant Plasmodium Infection in Mobile Populations Crossing Cambodian Borders
Source: PLoS One. 2015 Sep 9;10(9):e0124300. doi: 10.1371/journal.pone.0124300 (PMC4564195; doi:10.1371/journal.pone.0124300)
Supplement: S3 Table — Variables listed are those which were retained in the final multivariate model, with both the output from univariate (crude OR) and multivariate (adjusted OR) models presented with odds ratios (OR) and 95% confidence intervals (95% CI), as well as likelihood ratio test p values. OR = Odds ratio CI = Confidence interval DK = Don't know Forest-goer refers to someone who slept overnight in the forest at least once in the previous 6 months. (DOCX) [file pone.0124300.s003.docx]

**Table S3. Main risk factors for *Plasmodium vivax* infection (both symptomatic and asymptomatic) identified by RT-PCR analysis. Variables listed are those which were retained in the final multivariate model, with both the output from univariate (crude OR) and multivariate (adjusted OR) models presented with odds ratios (OR) and 95% confidence intervals (95% CI), as well as likelihood ratio test p values.**

| **Variable** | | **Positivity rate (%)** | **Crude OR (95% CI)** | **p-value** | **Adjusted OR (95% CI)** | **p-value** |
| --- | --- | --- | --- | --- | --- | --- |
|  |  |  |  |  |  |  |
| **Sex** | **Male** | 4.74 | 3.63 (2.06-6.37) | <0.0001 | 1.47 (0.79-2.71) | 0.2 |
|  | **Female** | 1.36 | 1 |  | 1 |  |
|  |  |  |  |  |  |  |
| **Age (years)** | **<15** | 2.28 | 1 | 0.3 | 1 | 0.9 |
|  | **15-40** | 3.93 | 1.75 (0.70-4.36) |  | 0.87 (0.34-2.27) |  |
|  | **>40** | 3.21 | 1.42 (0.53-3.76) |  | 0.82 (0.30-2.26) |  |
|  |  |  |  |  |  |  |
| **Time of crossing** | **am** | 2.87 | 1 |  | 1 |  |
|  | **pm** | 6.87 | 2.50 (1.70-3.67) | <0.0001 | 1.83 (1.20-2.78) | 0.006 |
|  |  |  |  |  |  |  |
| **Frequency of crossing** | **≥once per week** | 2.25 | 1 |  | 1 |  |
|  | **<once per week** | 5.16 | 2.37 (1.54-3.63) | <0.0001 | 1.45 (0.92-2.29) | 0.007 |
|  | **DK** | 1.13 | 0.50 (0.15-1.65) |  | 0.35 (0.10-1.18) |  |
|  |  |  |  |  |  |  |
| **Fever (≥37.5^o^C)** | **No** | 3.13 | 1 | <0.0001 | 1 | 0.02 |
|  | **Yes** | 11.17 | 3.88 (2.40-6.28) |  | 1.92 (1.13-3.27) |  |
|  |  |  |  |  |  |  |
| **Previous malaria episode** | **Yes** | 7.02 | 5.29 (3.38-8.29) | <0.0001 | 2.97 (1.82-4.83) | <0.0001 |
|  | **No** | 1.41 | 1 |  | 1 |  |
|  | **Don't know** | 0.85 | 0.60 (0.08-4.46) |  | 0.50 (0.07-3.78) |  |
|  |  |  |  |  |  |  |
| **Forest-goer** | **No** | 1.91 | 1 | <0.0001 | 1 | <0.0001 |
|  | **Yes** | 10.14 | 6.13 (4.17-8.99) |  | 3.02 (1.93-4.70) |  |
|  |  |  |  |  |  |  |
| OR = Odds Ratio  CI = Confidence Interval  DK = Don't know  Forest-goer refers to someone who slept overnight in the forest at least once in the previous 6 months. | | | | | | |
